# Utilizing biologic disease-modifying anti-rheumatic treatment sequences to subphenotype rheumatoid arthritis

**DOI:** 10.1186/s13075-023-03072-0

**Published:** 2023-06-02

**Authors:** Priyam Das, Dana Weisenfeld, Kumar Dahal, Debsurya De, Vivi Feathers, Jonathan S. Coblyn, Michael E. Weinblatt, Nancy A. Shadick, Tianxi Cai, Katherine P. Liao

**Affiliations:** 1 Department of Biomedical Informatics, Harvard Medical School, Boston, MA USA; 2 Department of Biostatistics, Virginia Commonwealth University, Richmond, VA USA; 3 Division of Rheumatology, Inflammation, and Immunity, Brigham and Women’s Hospital, 60 Fenwood Road, Boston, MA 02115 USA; 4 Indian Statistical Institute, Kolkata, India

**Keywords:** Rheumatoid arthritis, Medication prescriptions, Biologic disease-modifying anti-rheumatic drugs, Electronic health record, Mixture model, Markov chain

## Abstract

**Background:**

Many patients with rheumatoid arthritis (RA) require a trial of multiple biologic disease-modifying anti-rheumatic drugs (bDMARDs) to control their disease. With the availability of several bDMARD options, the history of bDMARDs may provide an alternative approach to understanding subphenotypes of RA. The objective of this study was to determine whether there exist distinct clusters of RA patients based on bDMARD prescription history to subphenotype RA.

**Methods:**

We studied patients from a validated electronic health record-based RA cohort with data from January 1, 2008, through July 31, 2019; all subjects prescribed ≥ 1 bDMARD or targeted synthetic (ts) DMARD were included. To determine whether subjects had similar b/tsDMARD sequences, the sequences were considered as a Markov chain over the state-space of 5 classes of b/tsDMARDs. The maximum likelihood estimator (MLE)-based approach was used to estimate the Markov chain parameters to determine the clusters. The EHR data of study subjects were further linked with a registry containing prospectively collected data for RA disease activity, i.e., clinical disease activity index (CDAI). As a proof of concept, we tested whether the clusters derived from b/tsDMARD sequences correlated with clinical measures, specifically differing trajectories of CDAI.

**Results:**

We studied 2172 RA subjects, mean age 52 years, RA duration 3.4 years, and 62% seropositive. We observed 550 unique b/tsDMARD sequences and identified 4 main clusters: (1) TNFi persisters (65.7%), (2) TNFi and abatacept therapy (8.0%), (3) on rituximab or multiple b/tsDMARDs (12.7%), (4) prescribed multiple therapies with tocilizumab predominant (13.6%). Compared to the other groups, TNFi persisters had the most favorable trajectory of CDAI over time.

**Conclusion:**

We observed that RA subjects can be clustered based on the sequence of b/tsDMARD prescriptions over time and that the clusters were correlated with differing trajectories of disease activity over time. This study highlights an alternative approach to consider subphenotyping of patients with RA for studies aimed at understanding treatment response.

## Background

Over the past decade, the increasing number of biologic disease-modifying anti-rheumatic drugs (bDMARDs) and targeted synthetic DMARDs (tsDMARDs) has expanded the options that allow for effective treatment of patients with rheumatoid arthritis (RA). However, knowing which bDMARD or tsDMARD would be most effective for a particular patient remains an area of active investigation [1, 2]. The majority of studies have focused on phenotyping patients based on response to the most common bDMARD, tumor necrosis factor inhibitors (TNFi) [3, 4]. The current options for RA therapy allow us to reconsider ways to study patient subgroups based on the sequence of therapies they have used to control RA beyond TNFi [5, 6]. However, each patient’s treatment history is unique leading to hundreds or thousands of different medication sequences in a given RA cohort. Recent advances in biostatistics methods can be applied to cluster patients with similar sequences which can serve as an alternative approach to subphenotyping RA.

Six main classes of bDMARD and tsDMARDs targeting TNFi, CTLA4, interleukin (IL)-1, IL-6, Janus kinase (JAK), and CD20 are used to treat RA. The first TNFi, etanercept, was approved for RA in the USA in 1998. By 2008, patients and their rheumatologists had options for 3 of the 5 classes. TNFis remain the most commonly prescribed bDMARD in the USA and are usually the first drug prescribed after inadequate response to first-line therapy. A prior study found that most RA patients undergo changes in therapies due to loss of efficacy, with 50% discontinuing their first bDMARD after the first 24 months [7]. The majority of studies on treatment response in RA center around TNFi and whether subjects responded to TNFi in a defined period of time [8–10]. The current breadth of RA therapies available provides options for studies of RA patients who persist on TNFi despite alternative options vs those who undergo trials of multiple classes of DMARDs. However, the challenge is defining the different groups beyond those who persist on TNFi.

Few studies characterize RA patients based on their treatment history. Due to the complexity of these data, it is difficult to determine if patients are similar based on their past b/tsDMARD use. Bioinformatics methods are now available that can cluster sequences by similarity [11]. Thus, the objective of this study is to adapt methods using Markov chains for clustering sequence data to group RA subjects by the sequence of medications tried. By leveraging data from a linked RA prospective cohort study, we additionally test whether these clusters correlate with different RA clinical factors over time.

## Methods

### Study population

We utilized data from an electronic health record (EHR)-based cohort of RA subjects classified using one RA ICD code from two large tertiary care centers, Brigham and Women’s Hospital and Massachusetts General Hospital [12, 13]. We included subjects who had a b/tsDMARD prescription on or after January 1, 2008, with no RA prescriptions prior to 2008, an RA International Classification of Diseases (ICD) code ≥ 3 months prior to the b/tsDMARD start date, and two b/tsDMARD prescriptions ≥ 3 months apart. By 2008, multiple b/tsDMARD options were available beyond TNFi and electronic prescriptions were mandated at our institution, enabling more complete capture of medication data. Data on prescriptions were extracted up to July 31, 2019. The RA ICD code requirement decreased the likelihood that subjects entered the hospital system already on a b/tsDMARD. A subset of these subjects were also followed as part of the Brigham Rheumatoid Arthritis Sequential Study (BRASS), a prospective longitudinal registry where RA clinical data such as disease activity, e.g., clinical disease activity index (CDAI), were collected at regular intervals during study visits [14]. We additionally performed a sensitivity analysis, eliminating the RA ICD code requirement prior to the 1st b/tsDMARD prescription to allow more subjects to be included.

### Mixture Markov model

Markov chains are one of the most well-known and widely used discrete time state space models [15]. Notable applications of the mixture Markov model can be found in the field of music analysis [11] and for identifying types of listeners using music station data [16]. While Markov chains have been recently applied to longitudinal clinical cohort data [17], to our knowledge, the mixture Markov model has not been used for EHR data characterization and analysis and is thus one of the objectives of this study. In the present study, we clustered RA patients based on their b/tsDMARD medication sequence. To do so, we considered the sequences to arise from a mixture of Markov chains where initially the number of clusters is unknown.

### Method to assign sequences to a cluster

After extracting b/tsDMARD prescriptions for all subjects, each subject had a medication sequence defined by their EHR data. As per the Markov chain methods, we assumed that each subject’s sequence of medications emerged from an unknown but finite number of clusters; the characteristics of the treatment sequence a subject has undergone depend on the cluster the subject belongs to. After maximizing the obtained likelihood (under the assumption that the treatment sequences are coming from a mixture of Markov chains) for different possible number of clusters, the true number of clusters can be estimated using the Akaike information criterion (AIC) and the Calinski-Harabasz (C-H) score. Once the number of clusters is estimated, we can then apply the maximum likelihood estimate (MLE) of the model parameters for that particular number of clusters. As a result, we can calculate the probability of any given treatment sequence belonging to each cluster. Treatment sequences are then assigned to the cluster where it has the highest probability of belonging. Thus, after estimating the parameter values of the proposed model, patients can be divided into a finite number of clusters, e.g., 4 clusters.

### Statistical analysis

We consider each b/tsDMARD sequence of RA patients as coming from an unknown number of Markov chains where state-space is given by 5 classes of the most commonly used b/tsDMARDs, namely TNFi (adalimumab, certolizumab, etanercept, infliximab, golimumab), CTLA4-Ig (abatacept), IL6R blockade (tocilizumab, sarilumab), JAK inhibitor (tofacitinib, baricitinib, upadacitinib), and Anti-CD20 (rituximab). Sequences were constructed by starting with the date of the first b/tsDMARD prescription and looking forward in 3-month periods. Each 3-month period was then divided into zero, single, or multiple encounters using the following rules:

1. Zero encounters: no prescribed b/tsDMARD therapy
2. Single encounter: if only one b/tsDMARD therapy was used. For example, if within the 3-month period, there were two prescriptions for drug B; then, the 3-month period would be described simply as B.
3. Multiple encounters: more than one b/tsDMARD was prescribed and consecutive drug encounters were combined. For example, within a 3-month period, the drug sequence A ➔ A ➔ C ➔C would be reconstructed as A ➔ C.

Only one b/tsDMARD was prescribed in the majority of the 3-month windows. An example sequence for an RA patient over the state-space of b/tsDMARDs is Encounter 1, TNFi ➔Encounter 2, TNFi ➔Encounter 3, TNFi ➔Encounter 4, tocilizumab ➔Encounter 5, abatacept.

As stated above, we first assigned medication sequences for each subject based on their prescriptions. Next, the sequences were assigned probabilities of belonging to a potential cluster. To estimate the number of clusters in the entire cohort, a mixed Markov model was fitted for *K* = 2, 3, 4, 5 components. The true number of clusters was estimated using AIC and the C-H score [18]. Based on the lowest AIC and the highest C-H score (of yearly rate of b/tsDMARDs for different possible number of clusters), the optimal number of clusters observed in our dataset was *K* = 4. Once the number of clusters was estimated, we applied the MLE of the model parameters for the 4 clusters. We then calculated the probability of any given treatment sequence belonging to each cluster and assigned each sequence to the cluster where it had the highest probability, resulting in individual patients assigned to one of 4 clusters.

### Association with clinical factors

Clinical data were extracted from the EHR including age, self-reported race and ethnicity, electronic medication prescriptions, comorbidities based on ICD codes, and seropositivity, defined as a positive result for either rheumatoid factor (RF) or anti-cyclic citrullinated peptide (anti-CCP) antibodies. RA follow-up time prior to 1st b/tsDMARD was defined as the date of a subject’s 1st RA ICD code to the date of their 1st b/tsDMARD electronic prescription. Follow-up time was defined as the date of their 1st b/tsDMARD prescription to their last encounter in the EHR. Means and standard deviations were used to summarize normally distributed continuous variables, medians, and IQRs were used for variables with non-nor, l distributions, and percent was used to summarize categorical variables. For categorical and normally distributed continuous variables, we used one-way ANOVA to test for differences in proportions or mean values across the medication clusters; the Kruskal–Wallis test was used for non-normally distributed continuous variables. Post hoc testing was done for all variables that had significant differences across clusters. The Tukey post hoc test for multiple comparisons of means followed the one-way ANOVAs and the Dunn test was used following the Kruskal–Wallis test.

To examine the correlation of these clusters to established clinical correlates, we linked the EHR RA cohort with the longitudinal BRASS registry which contains prospective collected data on RA disease activity. For each BRASS subject, we calculated their mean CDAI for each year, starting with the year prior to their 1st b/tsDMARD. We plotted the mean CDAI for each cluster in the year prior to b/tsDMARD start and the following 6 years. CDAI was used to measure disease activity because it does not include CRP, which can be directly impacted by IL-6 blockers, one of the major b/tsDMARD classes. Alternative disease activity measures using ESR were not available because ESR was not collected as part of the BRASS registry. We further compared differences between TNFi persisters (cluster 1) vs subjects who used alternate or multiple b/tsDMARDs (clusters 3 and 4).

This study was reviewed and approved by the Mass General Brigham Institutional Review Board. Patient consent was waived by the approving ethics committee. All analyses were performed using R 3.6.3 and Matlab R2019b [19].

## Results

In total, 5570 subjects had a new bDMARD or tsDMARD prescription in 2008 or later, comprising 78,792 unique encounters and 19,021 total 3-month periods. Of those subjects, 2951 had an RA ICD code ≥ 3 months prior to their first b/tsDMARD and 2172 also had ≥ 2 b/tsDMARD prescriptions 3 months apart. The 2172 patients in the primary analysis cohort had 37,935 distinct prescriptions and 550 unique sequences. The cohort had mean age 52, 76% female, 83% White, 6.8% Black, and 4.6% Hispanic, and 62% seropositive. The median RA duration was 1.7 years. During the study period, TNFi was the most common 1st b/tsDMARD (81%), followed by rituximab (8.9%), and abatacept (5.6%); 3.0% initiated an IL-6 blocker and 1.2% a JAK inhibitor.

**Fig. 1:**
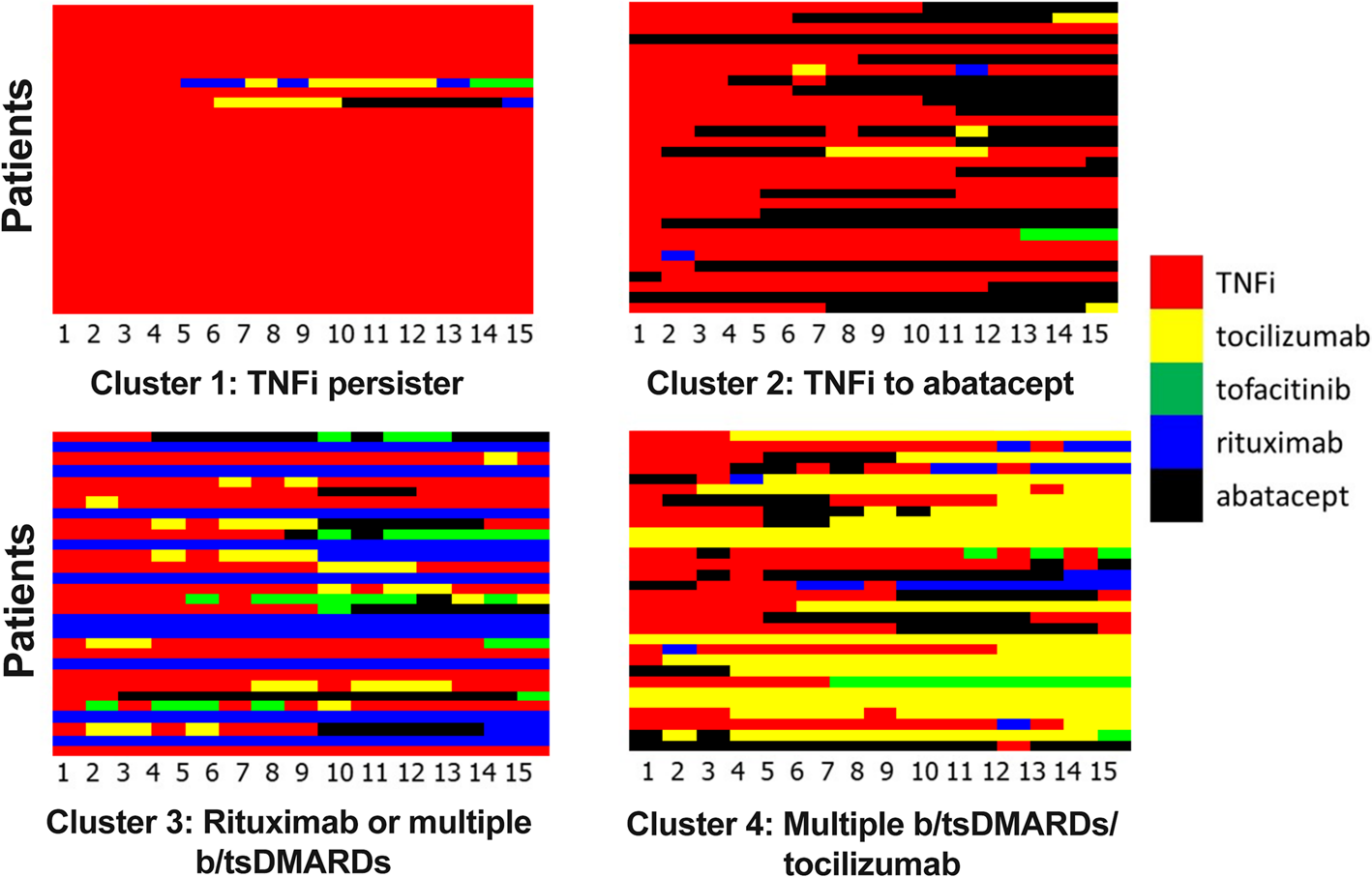
Medication sequences for a random 30 RA subjects from each cluster with ≥ 15 sequences. Only the first 15 sequences (3.75 years of medication data) are shown, even if the subject had additional bDMARD prescriptions

The optimal number of clusters was identified to be 4 using AIC and the C-H score. Nearly two thirds of subjects were in cluster 1, consisting mainly of TNFi persisters, *n* = 1,427 (66%). Cluster 4, subjects on multiple bDMARDs enriched for tocilizumab, and cluster 3, subjects on rituximab or multiple bDMARDs, had similar numbers, *n* = 296 (14%) and *n* = 275 (13%), respectively. Cluster 2, TNFi to abatacept, had the fewest subjects, *n* = 174 (8.0%) (Fig. 1). The clusters were named based on the predominant treatment pattern within the cluster. The expanded analysis including all 5570 subjects with first bDMARD and RA medication prescription on or after 2008 also identified the same 4 clusters with subjects assigned in similar proportions; cluster 1, TNFi persisters, 67%; cluster 2, TNFi/abatacept, 5.7%; cluster 3, rituximab/multiple bDMARDs, 13%; cluster 4, multiple bDMARDs/tocilizumab, 13%.

In cluster 1, TNFi persisters, 96% of subjects were only prescribed TNFi. In cluster 2, 59% of subjects started on TNFi and 34% started on abatacept, with 76% on abatacept at their last encounter date. Cluster 3 consisted mainly of subjects who were only prescribed rituximab (63%); the remaining subjects were all prescribed > 1 medication class. Cluster 4 had the greatest variety in medication classes prescribed as well as the largest proportion of subjects who were prescribed > 1 b/tsDMARD class, with 60% trying 2 classes and 21% trying 3 to 5 classes. Cluster 4 had 64% of subjects start on TNFi, 19% on tocilizumab, 12% on abatacept, and 4% on JAKi; 35% ended on tocilizumab, 20% on rituximab, 18% on JAKi, 16% on TNFi, and 10% on abatacept.

**Fig. 2:**
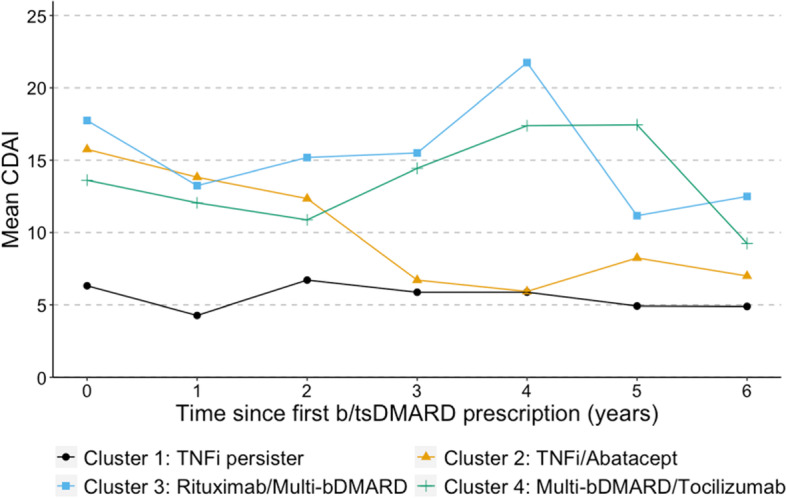
Comparison of mean CDAI across 4 clusters among subjects using linked EHR and RA registry data for 6 years following first b/tsDMARD prescription

There were significant differences in clinical characteristics across the clusters, including age, sex, RA duration, and seropositivity rate (Table 1). The TNFi persister cluster had a significantly lower seropositivity rate and younger age at first b/tsDMARD than the other three clusters and a higher percentage of male subjects than the TNFi/abatacept and multi-bDMARD/tocilizumab groups. At the time of first biologic or targeted synthetic DMARD, the TNFi persister cluster had the lowest average CDAI, which remained in the low disease activity range after initiating biologic therapy (Fig. 2). The other three clusters had moderate to high disease activity at the time of first b/tsDMARD. The two multiple b/tsDMARD clusters had unstable moderate to high disease activity over time. In contrast, the TNFi/abatacept group had a downward sloping directory, stabilizing with low disease activity after several years.

**Table 1:** Comparison of baseline clinical characteristics of RA subjects at the time of their 1st b/tsDMARD across medication sequence clusters

	Cluster 1: TNFi persister	Cluster 2: TNFi/abatacept	Cluster 3: Rituximab/multiple bDMARD	Cluster 4: Multiple bDMARD/tocilizumab	*P*-value
Age at 1st biologic, mean (SD)	51 (16)	54 (15)	57 (15)	55 (14)	< 0.001
Female	74%	87%	83%	77%	< 0.001
Race					0.104
White	84%	77%	83%	84%	
Black	5.8%	11%	9%	7.3%	
Other	11%	13%	8.5%	9.1%	
Hispanic	4.4%	8.0%	3.6%	4.4%	0.143
RA follow-up^a^, median years [IQR]	1.5 [0.65, 4.5]	1.8 [0.88, 3.5]	2.9 [0.81, 6.2]	1.5 [0.57, 4.8]	< 0.001
Follow-up time^b^, median years [IQR]	5.2 [3.0, 7.3]	5.8 [3.9, 7.7]	4.2 [2.4, 6.6]	4.7 [2.8, 7.3]	< 0.001
Seropositivity	57%	72%	73%	69%	< 0.001

^a^RA follow-up time prior to 1st b/tsDMARD

^b^Follow-up time from 1st b/tsDMARD to last encounter in the EHR

## Discussion

In this study, we applied an approach that allowed us to group RA subjects with similar bDMARD and tsDMARD prescription medication sequence over time, identifying 4 clusters. The largest cluster was TNFi persisters, followed by those who were prescribed rituximab or multiple b/tsDMARDs and b/tsDMARD cyclers remaining largely on tocilizumab. The smallest cluster comprised a large proportion of subjects on abatacept or on TNFi who switched to and remained on abatacept. Furthermore, after linking this cluster data with an RA registry with prospectively collected RA disease activity data, we demonstrated that these clusters were correlated with clinical outcomes. The clusters were associated with different RA disease activity starting points and trajectories over time. The TNFi persisters correlated with a subgroup of RA subjects who had the best trajectory for disease activity control, followed by those who remained on abatacept. As anticipated, those who required trials of multiple b/tsDMARDs had the least control of RA disease activity.

TNFis are the most commonly prescribed bDMARD in the USA owing to the availability with 5 formulations, with the approval of the first TNFi in 1998. Since it is also commonly the first drug prescribed, TNFi persisters unsurprisingly comprise the largest group. In clinical practice, studies have shown that abatacept and rituximab are more effective among seropositive subjects [20]. Our real-world data findings are in line with these findings where a high percentage of seropositive subjects who do not have good RA disease control on TNFi do eventually remain on abatacept and rituximab.

While the correlation between the medication sequence clusters with RA disease activity over time was anticipated, it enabled confirmation of the clinical relevance of these clusters determined using b/tsDMARD medication history alone. Thus, these results highlight a potential role in using these sequences as a proxy for disease activity trajectories or subgroups of patients with difficult to treat RA for further study [21]. RA disease activity scores require careful documentation of elements such as swollen and tender joints and the patient global which are not routinely collected in clinical care. In this study, we leveraged BRASS, which carefully collected these data longitudinally since 2003. In datasets which may have detailed medication data, the medication clusters can potentially provide information regarding RA disease control. Work is underway to extract more granular data from the EHR to determine if differing baseline characteristics can predict the probability of an individual belonging to a cluster in the future.

The grouping of clusters around the different targets, e.g., TNFi and IL-6, vs no distinct clusters or highly mixed clusters suggests that subjects may be grouped by the dominant pathway driving their RA disease activity. However, defining the clusters using EHR data represents only one of several key steps towards translating these data for use in the clinical setting. Future directions include determining whether these clusters correlate with biology, e.g., whether subjects within each cluster have more similar underlying genetics compared to across clusters. Genetics in turn may be used to inform earlier which patient is more likely to respond to which treatment.

While Markov chains have been recently applied to longitudinal clinical cohort data [17], to our knowledge, mixture Markov modeling has not been used for EHR data characterization and analysis. This study provides a roadmap application of this method across other clinical conditions where multiple treatments are available.

Limitations of this study include that the EHR cohort used data derived from two large tertiary care centers which may not be representative of general rheumatology practices. While we required restrictions to define RA beyond > 1 RA ICD code, e.g., requirement of > 1 RA ICD in the 6 months prior to b/tsDMARD prescription, misclassification of subjects remains a possibility. There are also circumstances where electronic prescriptions are ordered but the prescription is not ultimately filled. This scenario would introduce misclassification and could result in reduced power to detect differences across clusters. Additionally, b/tsDMARD options are impacted by insurance coverage, which could limit the variation of sequences, and reduce the number of potential clusters, as well as our ability to detect relationships between clinical factors and the clusters. Finally, while we attempted to minimize this with our inclusion criteria, subjects may have been prescribed b/tsDMARDs prior to their first visit at our centers. The sensitivity analysis without this additional filter resulted in a similar 4 clusters, supporting the robustness of our findings.

## Conclusion

We applied a mixture Markov model and identified 4 distinct clusters based on the sequence of bDMARD and tsDMARD medications used by subjects with RA ICD codes: (1) TNFi persisters, (2) TNFi and abatacept therapy, (3) rituximab or multiple b/tsDMARDs, and (4) multiple therapies with tocilizumab predominant. We demonstrated proof of concept that these clusters correlated with differing clinical disease activity trajectories. As more data become available to consider for studies on RA treatment response, this study highlights the role of alternative types of data, in this case prescription b/tsDMARD medication sequences as an approach to subphenotype patients with RA using EHR data. Future directions include examining whether the subphenotypes defined by their b/tsDMARD use also have distinct genomic or biomarker signatures in this and other cohorts, to inform our understanding of treatment response in RA.

## Data Availability

The datasets generated and used for this study are available on request from the corresponding author, KPL, and upon approval from the institutional ethical review board.

## Abbreviations

AIC: Akaike information criterion
bDMARD: Biologic disease-modifying anti-rheumatic drug
BRASS: Brigham Rheumatoid Arthritis Sequential Study
C-H: Calinski-Harabasz
CCP: Cyclic citrullinated peptide
CDAI: Clinical disease activity index
EHR: Electronic health record
ICD: International Classification of Diseases
IL: Interleukin
JAK: Janus kinase
MLE: Maximum likelihood estimate
RA: Rheumatoid arthritis
RF: Rheumatoid factor
TNFi: Tumor necrosis factor inhibitor
tsDMARD: Targeted synthetic disease-modifying anti-rheumatic drug

## References

[1] Holdsworth EA, Donaghy B, Fox KM, Desai P, Collier DH, Furst DE (2021). Biologic and targeted synthetic DMARD utilization in the United States: Adelphi Real World disease specific programme for rheumatoid arthritis. Rheumatol Ther.

[2] Aletaha D, Smolen JS (2018). Diagnosis and management of rheumatoid arthritis. JAMA.

[3] Frisell T, Baecklund E, Bengtsson K, Di Giuseppe D, Forsblad-D’Elia H, Askling J (2018). Patient characteristics influence the choice of biological drug in RA, and will make non-TNFi biologics appear more harmful than TNFi biologics. Ann Rheum Dis.

[4] Mian AN, Ibrahim F, Scott IC, Bahadur S, Filkova M, Pollard L (2016). Changing clinical patterns in rheumatoid arthritis management over two decades: sequential observational studies. BMC Musculoskelet Disord.

[5] Fraenkel L, Bathon JM, England BR, St.Clair EW, Arayssi T, Carandang K (2021). 2021 American College of Rheumatology guideline for the treatment of rheumatoid arthritis. Arthritis Care Res (Hoboken).

[6] Zhao SS, Kearsley-Fleet L, Bosworth A, Watson K, Hyrich KL (2022). Effectiveness of sequential biologic and targeted disease modifying anti-rheumatic drugs for rheumatoid arthritis. Rheumatology.

[7] Strand V, Miller P, Williams SA, Saunders K, Grant S, Kremer J (2017). Discontinuation of biologic therapy in rheumatoid arthritis: analysis from the Corrona RA registry. Rheumatol and Ther.

[8] Law-Wan J, Sparfel MA, Derolez S, Azzopardi N, Goupille P, Detert J (2021). Predictors of response to TNF inhibitors in rheumatoid arthritis: an individual patient data pooled analysis of randomised controlled trials. RMD Open.

[9] Guan Y, Zhang H, Quang D, Wang Z, Parker SCJ, Pappas DA (2019). Machine learning to predict anti–tumor necrosis factor drug responses of rheumatoid arthritis patients by integrating clinical and genetic markers. Arthritis Rheumatol.

[10] Karlsson JA, Kristensen LE, Kapetanovic MC, Gulfe A, Saxne T, Geborek P (2007). Treatment response to a second or third TNF-inhibitor in RA: results from the South Swedish Arthritis Treatment Group Register. Rheumatology.

[11] Qi Y, Paisley JW, Carin L (2007). Music analysis using hidden Markov mixture models. IEEE Trans Signal Process.

[12] Liao KP, Cai T, Gainer V, Goryachev S, Zeng-Treitler Q, Raychaudhuri S (2010). Electronic medical records for discovery research in rheumatoid arthritis. Arthritis Care Res.

[13] Huang S, Huang J, Cai T, Dahal KP, Cagan A, He Z (2020). Impact of ICD10 and secular changes on electronic medical record rheumatoid arthritis algorithms. Rheumatology (Oxford).

[14] Iannaccone CK, Lee YC, Cui J, Frits ML, Glass RJ, Plenge RM (2011). Using genetic and clinical data to understand response to disease-modifying anti-rheumatic drug therapy: data from the Brigham and Women’s Hospital Rheumatoid Arthritis Sequential Study. Rheumatology (Oxford).

[15] Nicolas P. Understanding Markov chains: examples and applications. Numerical methods for partial differential equations. Singapore: Springer; 2013.

[16] Gupta R, Kumar R, Vassilvitskii S. On mixtures of markov chains. In: 30th conference on neural information processing systems. 2016. Available from: https://papers.nips.cc/paper/6078-on-mixtures-of-markov-chains.pdf. Cited 2022 Jan 30.

[17] de Haan-Rietdijk S, Kuppens P, Bergeman CS, Sheeber LB, Allen NB, Hamaker EL (2017). On the use of mixed Markov models for intensive longitudinal data. Multivariate Behav Res.

[18] Calinski T, Harabasz J (1974). A dendrite method for cluster analysis. Commun Stat Simul Comput.

[19] The MathWorks Inc. MATLAB version: 9.13.0 (R2022b). Natick: The MathWorks Inc.; 2022. https://www.mathworks.com.

[20] Alten R, Nüßlein HG, Mariette X, Galeazzi M, Lorenz HM, Cantagrel A (2017). Baseline autoantibodies preferentially impact abatacept efficacy in patients with rheumatoid arthritis who are biologic naïve: 6-month results from a real-world, international, prospective study. RMD Open.

[21] Nagy G, Roodenrijs NM, Welsing PM, Kedves M, Hamar A, van der Goes MC (2021). EULAR definition of difficult-to-treat rheumatoid arthritis. Ann Rheum Dis.

